# Supplementary material for: The Psychoemotional Stress-Induced Changes in the Abundance of SatIII (1q12) and Telomere Repeats, but Not Ribosomal DNA, in Human Leukocytes
Source: Genes (Basel). 2022 Feb 14;13(2):343. doi: 10.3390/genes13020343 (PMC8872136; doi:10.3390/genes13020343)

Flow Cytometry analysis (FCA)

Fixed cells were analyzed at CytoFLEX S (Beckman Coulter). The area of lymphocytes was determined on the plot SSC – FCS. The cells in that region are stained with antibodies to T-lymphocyte marker CD3. Primary data are presented as median values of the signal.

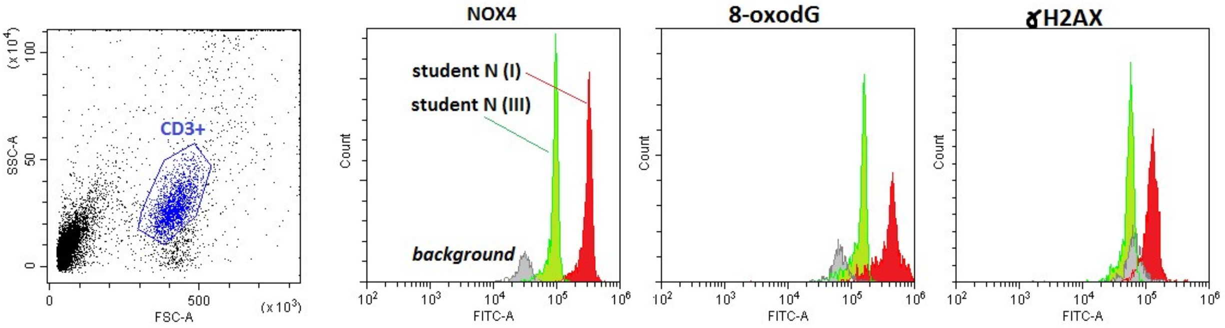

Supplement: Supplementary file 1 [file genes-13-00343-s001.zip › genes-1542400-supplementary.pdf]
